# Limited Value of Cystatin-C over Estimated Glomerular Filtration Rate for Heart Failure Risk Stratification

**DOI:** 10.1371/journal.pone.0051234

**Published:** 2012-12-11

**Authors:** Elisabet Zamora, Josep Lupón, Marta de Antonio, Joan Vila, Amparo Galán, Paloma Gastelurrutia, Agustín Urrutia, Antoni Bayes-Genis

**Affiliations:** 1 Heart Failure Unit, Hospital Universitari Germans Trias i Pujol, Badalona, Spain; 2 Department of Medicine, Autonomous University of Barcelona, Barcelona, Spain; 3 Inflammatory and Cardiovascular Disease Programme, IMIM-Hospital del Mar Research Institute, Barcelona, Spain; 4 CIBER Epidemiology and Public Health, Barcelona, Spain; 5 Biochemistry Service, Hospital Universitari Germans Trias i Pujol, Badalona, Spain; University of Virginia Health System, United States of America

## Abstract

**Background:**

To compare the prognostic value of estimated glomerular filtration rate, cystatin-C, an alternative renal biomarker, and their combination, in an outpatient population with heart failure.Estimated glomerular filtration rate is routinely used to assess renal function in heart failure patients. We recently demonstrated that the Cockroft-Gault formula is the best among the most commonly used estimated glomerular filtration rate formulas for predicting heart failure prognosis.

**Methodology/Principal Findings:**

A total of 879 consecutive patients (72% men, age 70.4 years [P_25–75_ 60.5–77.2]) were studied. The etiology of heart failure was mainly ischemic heart disease (52.7%). The left ventricular ejection fraction was 34% (P_25–75_ 26–43%). Most patients were New York Heart Association class II (65.8%) or III (25.9%). During a median follow-up of 3.46 years (P_25–75_ 1.85–5.05), 312 deaths were recorded. In an adjusted model, estimated glomerular filtration rate and cystatin-C showed similar prognostic value according to the area under the curve (0.763 and 0.765, respectively). In Cox regression, the multivariable analysis hazard ratios were 0.99 (95% CI: 0.98–1, *P* = 0.006) and 1.14 (95% CI: 1.02–1.28, *P* = 0.02) for estimated glomerular filtration rate and cystatin-C, respectively. Reclassification, assessed by the integration discrimination improvement and the net reclassification improvement indices, was poorer with cystatin-C (−0.5 [−1.0;−0.1], *P* = 0.024 and −4.9 [−8.8;−1.0], *P* = 0.013, respectively). The value of cystatin-C over estimated glomerular filtration rate for risk-stratification only emerged in patients with moderate renal dysfunction (eGFR 30–60 ml/min/1.73 m^2^, chi-square 12.9, *P*<0.001).

**Conclusions/Significance:**

Taken together, the results indicate that estimated glomerular filtration rate and cystatin-C have similar long-term predictive values in a real-life ambulatory heart failure population. Cystatin-C seems to offer improved prognostication in heart failure patients with moderate renal dysfunction.

## Introduction

Chronic heart failure (HF) is a growing public epidemic with increasing incidence and prevalence [1]. Despite important progress in recent decades, mortality remains high among patients with HF. Renal insufficiency is prevalent among patients with HF, and the coexistence of both conditions results in a worse prognosis [2]–[6]. The most precise methods for calculating kidney function, including the isotopic glomerular filtration rate and creatinine clearance in a 24-hour urine specimen, are not utilized in daily clinical practice [7]. Instead, several formulas based on creatinine clearance have been developed to determine the estimated glomerular filtration rate (eGFR), with the Cockroft-Gault formula [8], the simplified Modification of Diet in Renal Disease (MDRD-4) equation [9], and the Chronic Kidney Disease Epidemiology Collaboration equation [10] being the most commonly used in clinical practice. We recently demonstrated that the Cockroft-Gault formula is the best among these three eGFR formulas for predicting long-term prognosis in HF patients [11].

In the last few years, cystatin-C has emerged as a novel renal biomarker with prognostic implications in patients with HF [12]–[13]. However, to the best of our knowledge, no data have assessed the benefits of cystatin-C over eGFR in terms of prognosis in patients with chronic HF. The objective of the present study was to compare the long-term prognostic value of cystatin-C and eGFR using the Cockroft-Gault formula in an outpatient population with HF and to assess whether the simultaneous use of both markers is helpful in improving patient risk stratification.

## Methods

### Study Population

From May 2006 to July 2010, ambulatory patients treated at a multidisciplinary HF unit were consecutively included in the study. Patients were referred to the unit by cardiology or internal medicine departments and, to a lesser extent, from the emergency or other hospital departments. The principal referral criteria were HF according to the European Society of Cardiology guidelines irrespective of etiology, and at least one HF hospitalization and/or reduced left ventricular ejection fraction (LVEF). Blood samples were obtained by venipuncture between 9:00 a.m. and 12:00 p.m. during conventional ambulatory visits, and adequately centrifuged serum samples were stored at −80°C. Both cystatin-C and creatinine were analyzed from the same blood sample.

All participants provided written informed consent, and the local ethics committee approved the study. All study procedures were in accordance with the ethical standards outlined in the Helsinki Declaration of 1975 as revised in 1983.

### Follow-up and Outcomes

All patients were followed at regular predefined intervals with additional visits as required in the case of decompensation. The regular visitation schedule included a minimum of quarterly visits with nurses, biannual visits with physicians, and elective visits with geriatricians, psychiatrists, and rehabilitation physicians [11], [14]. Patients who did not attend the regular visits were contacted by telephone.

Death from all causes was the main outcome. Fatal events were identified from the clinical records of the HF unit, other hospital wards, the emergency room, general practitioners, and by contacting the patient's relatives. The data were verified using the databases of the regional and national health systems.

### Glomerular Filtration Rate

The eGFR was calculated using the Cockroft-Gault formula: (140-age in years)×weight in kilograms/(72×serum creatinine level in mg/dl) adjusted by sex (×0.85 in women) [8], and then adjusted by body surface area [11]. Serum creatinine levels were analyzed using the CREA method with a Dimension® Clinical Chemistry System (Siemens, Newark, USA) and a modification of the kinetic Jaffe reaction described by Larsen with picrate as the reactant.

### Cystatin-C

Cystatin-C was measured using a nephelometric technique that assesses immune complex formation between cystatin and antiserum anticystatin-C attached to latex particles. Assays were processed twice by a Delta nephelometer (ref. 010138; Radim SPA, Pomezia, Italy, ref NPP42). The coefficient of variation between assays was 2.9%. Normal values are 0.53–0.95 mg/L.

### Statistical Analysis

Categorical variables were expressed as percentages. Continuous variables were expressed as the mean (standard deviation) or median (25^th^ and 75^th^ percentiles [P_25–75_]) according to normal or non-normal distribution. Differences in cystatin-C levels between groups were compared using the Mann-Whitney and Kruskal Wallis tests, and correlations between cystatin-C and continuous variables were evaluated using the Rho Spearman coefficient. Colinearity between eGFR and Cystatin-C was assessed with Eigen-values analysis, Condition Index and Variance Inflation Factor.

Survival analyses were performed using Cox regression models incorporating the following variables: age, sex, New York Heart Association (NYHA) functional class, ischemic etiology of HF, LVEF (in %), HF duration, presence of diabetes mellitus, chronic obstructive lung disease and peripheral artery disease, plasma hemoglobin (g/dl), serum sodium (mmol/L), β-blocker treatment, and angiotensin-converting enzyme inhibitor (ACEI) or angiotensin II receptor blocker (ARB) treatment, together with eGFR (in ml/min/1.73 m^2^) or cystatin-C. A Cox regression model with both renal markers was also performed. Kaplan-Meier survival curves were plotted for eGFR and cystatin-C quartiles and the groups compared using the log-rank test. In addition, Kaplan-Meier survival curves were plotted for cystatin-C levels below or above the median for each quartile of eGFR.

We used different measurements of performance to test the potential incremental prognostic value of the two renal biomarkers.

- Discrimination: The area under the receiver operating characteristic curve (AUC) summarized the diagnostic discrimination. Discrimination refers to a model's ability to distinguish two classes of outcomes correctly. We used the index of rank correlation, Somers' D, which already incorporates information from censored data. AUCs between models were compared using the U-statistic test for equality concordance.
- Calibration: The D'Agostino–Nam version of the Hosmer and Lemeshow calibration test was used to calculate a chi-square value. A model is well calibrated when predicted and observed values agree for any reasonable grouping of the observation (no significant differences in the Hosmer–Lemeshow test). In addition, the Bayesian information criterion (BIC), the Akaike information criterion (AIC), and the Brier score were calculated for each model. Given any two estimated models, the model with the lower BIC, AIC, and Brier scores was preferred. No statistical tests compare different BIC, AIC, or Brier estimations, and lower values indicate a better model. When a biomarker was added to another, the global goodness-of-fit of the model was evaluated by a likelihood ratio test.
- Reclassification: We used the method described by Pencina et al [15]. Two main statistics are used to assess reclassification. The integrated discrimination improvement (IDI) considers changes in the estimated mortality prediction probabilities as a continuous variable. *P*-values less than 0.05 from two-sided tests were considered significant. The net reclassification improvement (NRI) requires a previous definition of meaningful risk categories (we used tertiles for the risk of death: <18.5%, 18.5–41%, and >41%). The NRI considers changes in the estimated mortality prediction probabilities that imply a change from one category to another.

All analyses were performed using the software R (version 2.11.1) statistical package (Foundation for Statistical Computing, Vienna, Austria).

## Results

Out of 891 consecutive patients included from May 22, 2006 to July 7, 2010, eGFR and cystatin-C were available in 879, which were finally included in this analysis. Median age of 70.4 years (P_25–75_ 60.5–77.2 years). Table 1 shows the baseline characteristics of the entire sample. During a median follow-up period of 3.46 years (P_25–75_ 1.85–5.05), 312 patients died. Among the cardiovascular causes of death (167), refractory HF was responsible in 90 (53.9%) patients, sudden death in 31 (18.5%) patients, and acute myocardial infarction in 15 (9.0%) patients. Two patients were lost to follow-up and adequately censored.

**Table 1:** Demographic and clinical baseline characteristics and treatments during follow-up.

	N = 879
Age, yr*	70.4 (60.5–77.2)
Males–no. (%)	631 (71.8)
White–no. (%)	874 (99.6)
Etiology–no. (%)	
Ischemic heart disease	463 (52.7)
Dilated cardiomyopathy	87 (9.9)
Hypertensive	80 (9.1)
Etoh	50 (5.7)
Toxic	23 (2.6)
Valvular	100 (11.4)
Other	76 (8.6)
HF duration, months*	26.9 (4–72)
LVEF, in %*	34 (26–43)
BMI, kg/m^2^ *	26.9 (24.2–30.5)
NYHA functional class–no. (%)	
I	65 (7.4)
II	578 (65.8)
III	228 (25.9)
IV	8 (0.9)
Hypertension–no. (%)	537 (61.1)
Diabetes mellitus–no. (%)	314 (35.7)
COLD–no. (%)	146 (16.6)
Treatments–no. (%)	
ACEI or ARB	791 (90.0)
β-blocker	771 (87.7)
Spironolactone/eplerenone	345 (39.2)
Loop diuretic	742 (84.4)
Digoxin	267 (30.4)
ICD	93 (10.6)
CRT	47 (5.3)
Sodium, mmol/L*	139 (137–142)
Hemoglobin, g/dl†	13.0±1.8
eGFR, ml/min/1.73 m^2^ *	42.4 (29.4–59.4)
Cystatin-C, mg/L*	1.21 (0.96–1.61)

* median (percentiles 25^th^ and 75^th^).

† (mean ± standard deviation).

ACEI, angiotensin-converting enzyme inhibitor; ARB, angiotensin II receptor blocker; BMI, body mass index; COLD, chronic obstructive lung disease; CRT, cardiac resynchronization therapy; eGFR, estimated glomerular filtration rate; Etoh, alcoholic cardiomyopathy; HF, heart failure; ICD, implantable cardiac defibrillator; LVEF, left ventricular ejection fraction; NYHA, New York Heart Association.

### Cystatin-C Levels

Cystatin-C levels correlated significantly with age (Rho 0.44, *P*<0.001) and eGFR (Rho −0.82, *P*<0.001), but not with LVEF (Rho 0.05, *P* = 0.12). However, no consistent colinearity was found between Cystatin-C and eGFR. Cystatin-C levels were significantly higher in women (*P* = 0.005), diabetic patients (*P*<0.001), hypertensive patients (*P*<0.001), and in patients not treated with β-blockers or ACEI-ARB. No relationship was found between cystatin-C levels and ischemic or non-ischemic HF etiology. In addition, cystatin-C levels progressively increased with worse NYHA functional class (*P*<0.001).

### Cox Regression and Modeling

In the bivariable analysis, both eGFR and cystatin-C predicted death from all causes as continuous variables (eGFR hazard ratio [HR] 0.97 [95% confidence interval (CI) 0.96–0.97], *P*<0.001; cystatin-C HR 1.30 [95% CI, 1.21–1.40], *P*<0.001). In separate multivariable analyses, both biomarkers remained independent predictors of mortality (Table 2). When both variables were incorporated into the multivariable analysis, a significant interaction was found (*P* = 0.001, Table 2), indicating that the effect of cystatin-C on prognosis differs according to eGFR.

**Table 2:** Multivariable Cox Regression analyses.

	Model with eGFR			Model with Cystatin-C			Model with eGFR, Cystatin-C and interaction eGFR^×^Cystatin-C		
	HR	95% CI	p-value	HR	95% CI	p-value	HR	95% CI	p-value
**Age**	1.05	[1.03;1.06]	<0.001	1.06	[1.04;1.07]	<0.001	1.05	[1.03;1.07]	<0.001
**Female gender**	0.75	[0.57;0.98]	0.036	0.74	[0.56;0.97]	0.031	0.75	[0.57;0.98]	0.037
**NYHA functional class**	1.74	[1.36;2.22]	<0.001	1.71	[1.33;2.18]	<0.001	1.65	[1.29;2.11]	<0.001
**Diabetes mellitus**	1.25	[0.99;1.57]	0.064	1.25	[0.99;1.58]	0.061	1.25	[0.99;1.58]	0.06
**Beta-blocker treatment**	0.5	[0.37;0.67]	<0.001	0.51	[0.38;0.7]	<0.001	0.49	[0.36;0.67]	<0.001
**ACEI or ARB treatment**	0.58	[0.42;0.79]	<0.001	0.59	[0.43;0.81]	0.001	0.58	[0.43;0.8]	<0.001
**LVEF**	0.99	[0.98;1]	0.057	0.99	[0.98;1]	0.032	0.99	[0.98;1]	0.05
**Ischemic aetiology of HF**	1.02	[0.8;1.3]	0.877	1.02	[0.8;1.3]	0.874	1.04	[0.81;1.33]	0.743
**HF duration**	1	[1;1]	0.044	1	[1;1]	0.043	1	[1;1]	0.062
**COLD**	1.14	[0.86;1.51]	0.349	1.16	[0.88;1.53]	0.303	1.1	[0.83;1.45]	0.524
**Peripheral artery disease**	1.52	[1.13;2.03]	0.005	1.51	[1.13;2.02]	0.006	1.48	[1.1;1.98]	0.009
**Na, mmol/L**	0.95	[0.92;0.98]	<0.001	0.95	[0.92;0.98]	0.001	0.95	[0.92;0.98]	<0.001
**Hb, g/dL**	0.88	[0.82;0.95]	<0.001	0.87	[0.81;0.93]	<0.001	0.9	[0.84;0.97]	0.005
**eGFR, ml/min/1.73m^2^**	**0.99**	**[0.98;1]**	**0.006**	-	-	-	**0.97**	**[0.96;0.99]**	**<0.001**
**Cystatin-C**	-	-	-	**1.14**	**[1.02;1.28]**	**0.02**	**0.89**	**[0.72;1.09]**	**0.249**
**Interaction eGFR^×^Cystatin-C**	-	-	-	-	-	-	**1.02**	**[1.01;1.03]**	**<0.001**

ACEI, angiotensin converting enzyme inhibitor; ARB, angiotensin II receptor blocker; BMI, body mass index; COLD, chronic obstructive lung disease; eGFR, estimated glomerular filtration rate; Hb, plasma hemoglobin; HF, heart failure; LVEF, left ventricular ejection fraction; Na, serum sodium; NYHA, New York Heart Association (I–II vs. III–IV).

**Figure 1:**
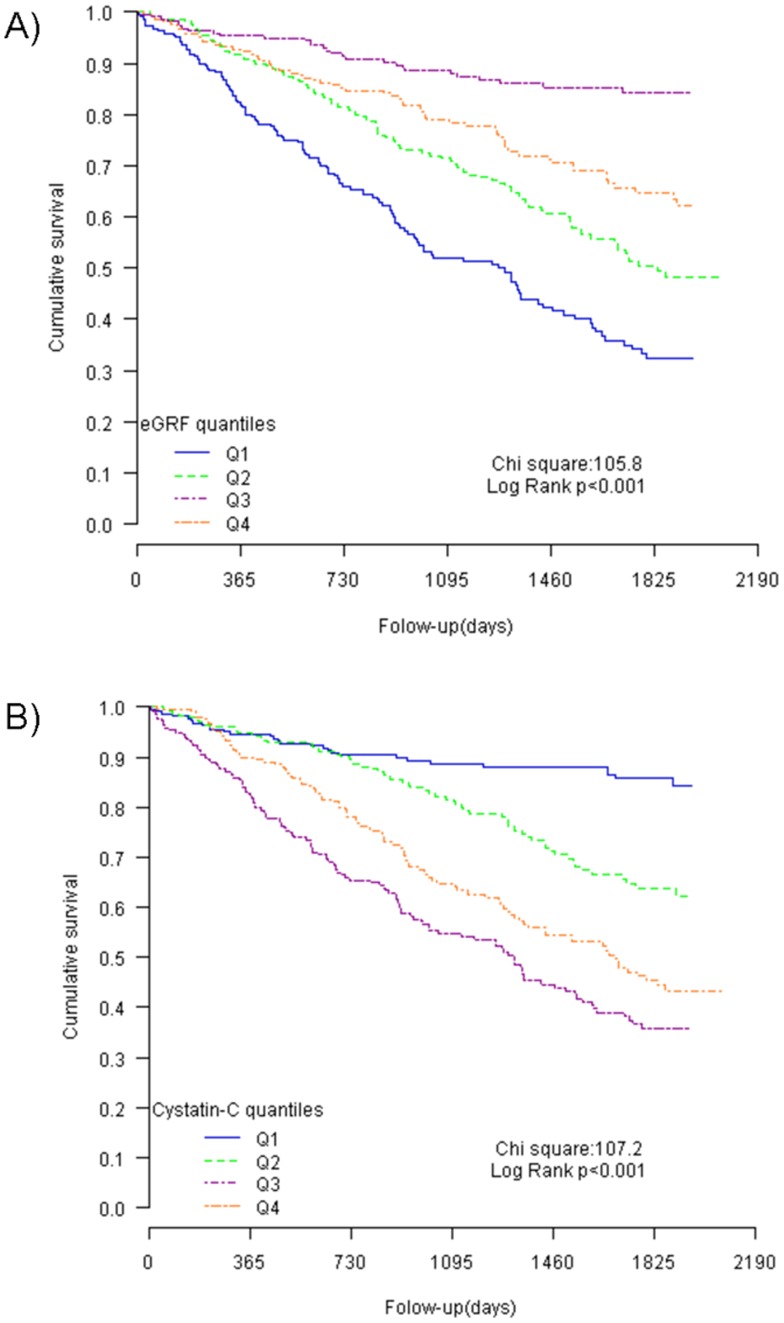
Kaplan–Meier survival curves according to eGFR and cystatin-C levels. Caption: Both eGFR levels (Panel A) and cystatin-C levels (Panel B) have been divided in quartiles.

**Figure 2:**
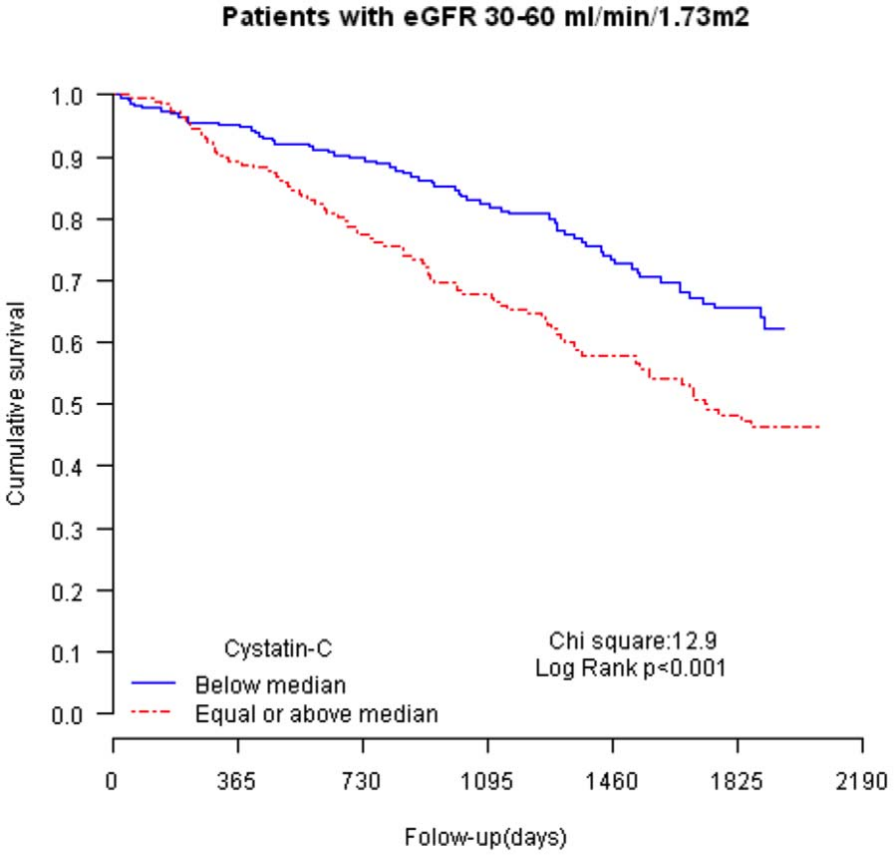
Kaplan–Meier survival curves according to cystatin-C levels in patients with an eGFR between 30 and 60 ml/min/1.73 m^2^. Caption: cystatin-C levels have been divided according to median values (below vs. equal/above). N = 443.

Kaplan–Meier survival curves according to eGFR (Figure 1A) and cystatin-C levels (Figure 1B) and divided into quartiles showed significant predictive prognostic values (log rank test chi-square 105.8 and 107.2; *P*<0.001 for both). When cystatin-C was analyzed as an addition to eGFR, its value for risk stratification was only present in moderate renal dysfunction patients (quartiles 2 and 3, eGFR 30–60 ml/min/1.73 m^2^, Figure 2).

### Measurements of Performance

#### eGFR vs. cystatin-C

The AUC for the prediction of death was very similar for eGFR and cystatin-C in the adjusted model (Table 3). The *P*-values for the Hosmer–Lemeshow statistics indicated good calibration for both markers (*P*>0.56 for all comparisons). Also BIC, AIC, and Brier scores were very similar for both markers (Table 3). Taking the model with eGFR as a reference, IDI (risk as a continuous variable) and NRI (reclassification according to predefined risk categories) decreased significantly with cystatin-C (−0.5 [−1.0;−0.1], *P* = 0.024 and −4.9 [−8.8;−1.0], *P* = 0.013, respectively) (Table 3).

**Table 3:** Performance of the adjusted models at 4 years.

	Model with eGFR	Model with Cystatin-C	Model with both eGFR and Cystatin-C	Model with eGFR, Cystatin-C and interaction eGFR^×^Cystatin-C
**AUC**	0.763 (0.737;0.79)	0.765 (0.739;0.791)	0.764 (0.738;0.79)	0.768 (0.742;0.794)
	reference	p-value: 0.577	p-value: 0.254	p-value: 0.159
**H-L**	Chi-square: 6.1	Chi-square: 7.7	Chi-square: 7.3	Chi-square: 4.4
	p.value = 0.73	p.value = 0.568	p.value = 0.608	p.value = 0.881
**Brier score**	0.16	0.16	0.159	0.157
**AIC**	3656.5	3659.4	3658.2	3648.5
**BIC**	3723.2	3726.1	3729.7	3724.8
**Likelihood R**	–	–	*p.value = 0.584	*p.value = 0.002
**IDI**		−0.5 [−1.0;−0.1],	−0.03 [−0.1;0.1],	1.0 [0.2;1.8],
	reference	*p-value = 0.024	*p-value = 0.619	*p-value = 0.01
**NRI-All**		−4.9 [−8.8;−1.0],	−2.0 [−3.9;−0.2],	2.4 [−2.6;7.5],
	reference	*p-value = 0.013	*p-value = 0.034	*p-value = 0.343
**NRI-Cases**		−3.1 [−5.9;−0.2],	−1.4 [−2.8;<0.1],	2.0 [−1.8;5.9],
	reference	*p-value = 0.033	*p-value = 0.045	*p-value = 0.298
**NRI-Control**		−1.8 [−4.2;0.6],	−0.6 [−1.8;0.6],	0.4 [−2.4;3.2],
	reference	*p-value = 0.133	*p-value = 0.336	*p-value = 0.783

* Versus model 1.

AUC, area under the ROC curve; AIC, Akaike information criterion; BIC, Bayesian information criterion; H-L, Hosmer and Lemeshow test; Likelihood R: Goodness-of-fit assessed by likelihood ratio; IDI, integrated discrimination improvement; NRI, net reclassification improvement.

Covariates included in models: Age, Female gender, NYHA functional class, Diabetes mellitus, Beta-blocker treatment, ACEI or ARB treatment, LVEF, Ischemic aetiology of HF, HF duration, COLD, Peripheral artery disease, Na, Hb.

#### Combined addition of eGFR and cystatin-C

The combined addition of the two markers in the adjusted model did not improve discrimination, calibration, or reclassification according to IDI and NRI (NRI was significantly worse: −2.0 [−3.9;−0.21], *P* = 0.034). However, when the variable interaction eGFR^×^cystatin-C was included in the model, the global goodness-of fit increased significantly (likelihood ratio *P*-value = 0.002) and reclassification using IDI significantly improved (1.0 [0.2;1.8], *P* = 0.01) with respect to the model with eGFR alone (Table 3), suggesting that cystatin-C affects prognosis according to eGFR.

## Discussion

Cystatin-C is a protein that belongs to a group of cysteine proteinase inhibitors, one of the four types of proteinases in mammalian cells. These types of proteins are encoded by the so-called housekeeping genes that regulate the factors necessary for global cell function, and all nucleated cells produce them at a stable production rate [16]. The protein is located extracellularly and detected mainly in biological fluids. Because of its small size, cystatin-C is freely filtered by the glomerulus and is not secreted, reabsorbed, or catabolized in the proximal tubules; it does not return to the blood and is not detected in urine [17]. Production depends on the metabolic rate and increases in hypermetabolic situations, such as hyperthyroidism and corticosteroid treatment [18]. Cystatin-C has been reported to provide a more accurate and precise estimate of GFR than serum creatinine [19]–[22]. In this study of a HF population, we found that cystatin-C levels were influenced by age, sex, NYHA functional class, eGFR, treatments, and comorbidities, such as diabetes and hypertension.

In recent years, cystatin-C has emerged as a marker of cardiovascular events and mortality in different situations. For example, in patients with ischemic heart disease, cystatin-C was found to be an independent risk factor together with traditional cardiovascular risk factors, renal function, or the presence of microalbuminuria [23]. The combined association of albuminuria and cystatin-C-based eGFR was associated with mortality, coronary heart disease, and HF outcomes in the ARIC community study [24]; and in the Cardiovascular Health Study it was a more powerful predictor of death and cardiovascular events in the elderly than creatinine [25]. Remarkably, the usefulness of cystatin-C as a cardiovascular-related prognostic biomarker has been linked not only to its ability to estimate renal function, but also to its relationship with ventricular remodeling and fibrosis and vascular wall stiffness [26]–[27].

In the specific setting of HF, most of the information on the prognostic usefulness of cystatin-C derive from acutely decompensated HF patients, and the data are encouraging. Lassus et al. [12] found that cystatin-C was a strong predictor of mortality in 480 patients hospitalized for acute HF, both in-hospital and during 1 year of follow-up, and was independent of other renal markers (serum creatinine and eGFR values estimated using the Cockroft-Gault formula). Interestingly, Naruse et al.[28] found the best relationship between high levels of cystatin-C and the risk of cardiac death in patients with acute HF and an eGFR calculated by the MDRD formula between 44 and 79 ml/min/1.73 m^2^, independent of volemia and body weight. Cystatin-C was also an independent predictor of prognosis at 2 years of follow-up for the occurrence of death, heart transplantation, or readmission due to worsening HF in 138 systolic HF patients admitted for acute descompensation [29].

In contrast, little information exists on the value of cystatin-C in chronic HF. The first work that examined the ability of cystatin-C to predict mortality in these patients was published by Shlipack et al. [13], who analyzed a subgroup of 279 patients with prevalent HF from the Cardiovascular Health Study. Cystatin-C was exclusively assessed by Cox regression analysis and remained a better independent pronosticator than creatinine and eGFR calculated by the simplified MDRD equation. Arimoto et al. [30], analyzing 140 patients with HF and 64 control subjects, found that serum cystatin-C levels were higher in the patients with HF. Patients with high cystatin-C levels had a markedly higher cardiac event rate (cardiac death and HF hospitalization), independent of creatinine levels. A recent publication [31] in a small cohort of 102 young patients with chronic HF assessed creatinine, eGFR calculated by MDRD and simplified MDRD formulas, and cystatin-C as predictors of renal function using isotope glomerular filtration rate as the gold standard. Despite the small number of recorded events (8 deaths, 10 HF hospitalizations, and 3 heart transplantations), cystatin-C levels were similar to both eGFR formulas for predicting renal function and had similar prognostic properties as MDRD and simplified MDRD in ROC analysis and Cox regression analysis. Our study included a substantially larger cohort of patients (879 vs. 102) who were older (median 70 years vs. mean 58 years), had greater impairment of renal function (eGFR 42.4 ml/min/1.73 m^2^ vs. 65 ml/min/1.73 m^2^), and higher cystatin-C levels (1.21 vs 0.8 mg/L). The follow-up also differed significantly (3.5 years vs. 2 years) and the number of deaths was much higher (312 vs. 8). The additional prognostic information gained by any marker over a clinical risk model plus other biomarkers needs to be determined using adequate statistical tools [32]; therefore we performed a very comprehensive state-of-the-art statistical analysis that included multivariate Cox regression, discrimination, calibration, and reclassification indices. In our study, cystatin-C had a similar predictive long-term prognostic value as eGFR estimated by the Cockroft-Gault formula after adjusting for some covariates according to discrimination, calibration, and Cox regression, though reclassication was poorer according to IDI and NRI.

Importantly, and not assessed in previous studies, we found that when both markers were used together, cystatin-C levels significantly affected prognosis, and differently according to eGFR. Remarkably, we found that cystatin-C improved risk stratification, mainly in patients with an eGFR between 30 and 60 ml/min/1.73 m^2^. This finding is in agreement with a previous study [28] in acutely decompensated HF patients (eGFR between 44 and 79 ml/min/1.73 m^2^).

Doubts still exist about the exact mechanism by which cystatin-C has predictive value in HF and whether its prognostic capacity goes beyond renal function. Damman et al. [31] studied the relationship between cystatin-C and inflammation; though they could not exclude some relationship between cystatin-C levels and several inflammatory markers, this effect seemed small in relation to the strong association between cystatin-C and glomerular filtration rate. Furthermore, whether the relationship of cystatin-C with the mechanisms of ventricular remodeling may influence its predictive role is unknown. Taking into account the reduced availability of cystatin-C in routine laboratories and the cost, its usefulness as a prognostic factor should only be considered in patients with moderate degrees of renal dysfunction and it is advisable to continue using the classical eGFR formulas.

This study has some limitations. The optimal time for determining cystatin-C in regards to the clinical situation and if it is better to make serial or a single determination are unknown. We have no data about the presence of hyperthyroidism, inflammatory parameters, or the use of corticosteroids, which may be related to metabolism and protease levels. Our population was a general HF population treated at a specific and multidisciplinary HF unit in a tertiary hospital; most patients were referred from the cardiology department and, accordingly, mainly experienced HF of ischemic etiology with reduced LVEF. As such, these results cannot necessarily be extrapolated to a global HF population.

## Conclusions

The eGFR and cystatin-C have a similar long-term prognostic value in ambulatory HF patients when analyzed in a model adjusted by several established mortality risk factors. Cystatin-C seems to offer improved prognostication in heart failure patients with moderate renal dysfunction.
